# TDP-43-mediated alternative polyadenylation is associated with a reduction in VPS35 and VPS29 expression in frontotemporal dementia

**DOI:** 10.1371/journal.pbio.3003573

**Published:** 2026-01-05

**Authors:** Vidhya Maheswari Jawahar, Yi Zeng, Ellen M. Armour, Mei Yue, Kathryn Citrano, Anastasiia Lovchykova, Madison M. Reeves, Bailey Rawlinson, Michael DeTure, Judith A. Dunmore, Yuping Song, Sophie K. Ball, Zbigniew K. Wszolek, Neill R. Graff-Radford, Bradley F. Boeve, David S. Knopman, Gregory S. Day, Scott A. Small, Dennis W. Dickson, Michael E. Ward, Tania F. Gendron, Yongjie Zhang, Mercedes Prudencio, Aaron D. Gitler, Leonard Petrucelli

**Affiliations:** 1 Department of Neuroscience, Mayo Clinic, Jacksonville, Florida, United States of America; 2 Department of Genetics, Stanford University School of Medicine, Stanford, California, United States of America; 3 Department of Neurology, Mayo Clinic, Jacksonville, Florida, United States of America; 4 Department of Neurology, Mayo Clinic, Rochester, Minnesota, United States of America; 5 Department of Neurology, Columbia University, New York, New York, United States of America; 6 Neuroscience Graduate Program, Mayo Clinic Graduate School of Biomedical Sciences, Jacksonville, Florida, United States of America; 7 National Institute of Neurological Disorders and Stroke, NIH, Bethesda, Maryland, United States of America; 8 Chan Zuckerberg Biohub—San Francisco, San Francisco, California, United States of America; Stony Brook University Medical Center: Stony Brook University Hospital, UNITED STATES OF AMERICA

## Abstract

TAR DNA-binding protein 43 (TDP-43) dysfunction is a hallmark of several neurodegenerative diseases, including frontotemporal dementia, amyotrophic lateral sclerosis, and Alzheimer’s disease. Although cryptic exon inclusion is a well-characterized consequence of TDP-43 loss of function, emerging evidence reveals broader roles in RNA metabolism, notably in the regulation of alternative polyadenylation (APA) of disease-relevant transcripts. In the present study, we examined 3′ untranslated region lengthening events in the brains of individuals with frontotemporal lobar degeneration with TDP-43 pathology (FTLD-TDP), focusing on the functional impact of APA dysregulation. To investigate whether TDP-43-mediated APA events occur in the postmortem brain, we measured the 3′ untranslated region length of the retromer component vacuolar protein sorting 35 (*VPS35*) and the ETS transcription factor (*ELK1*) in the frontal cortex of a large cohort of FTLD-TDP patients and of healthy controls, and evaluated if these APA events are associated with FTLD-TDP clinical characteristic, markers of TDP-43 pathology [e.g., hyperphosphorylated TDP-43 and cryptic stathmin-2 RNA], or the expression of VPS35 and VPS29 proteins, the latter being essential to the retromer complex. We identified robust 3′ untranslated region lengthening of *VPS35* and *ELK1* in FTLD-TDP, which strongly associated with markers of TDP-43 pathology, and *ELK1* APA also associated with an earlier age of disease onset. Functionally, *VPS35* APA was associated with reduced VPS35 and VPS29 protein expression, and lower VPS35 levels were associated with increased hyperphosphorylated TDP-43 and cryptic stathmin-2 RNA. Together, these data implicate APA dysregulation as a critical downstream consequence of TDP-43 dysfunction and suggest that TDP-43 loss may contribute to retromer impairment through APA-mediated repression of retromer subunits.

## Abstract 2

Recent work has shown that TDP-43 loss in frontotemporal dementia (FTD) induces changes in alternative polyadenylation, but the functional consequences of this are unclear. This study reports that 3′UTR lengthening of VPS35 in FTD patient brain samples correlates with reduced VPS35 and VPS29 protein levels, suggesting that TDP-43 loss induces retromer dysfunction.

## Introduction

Dysfunction of the TAR DNA binding protein 43 kDa (TDP-43) is implicated in several neurodegenerative diseases including frontotemporal dementia (FTD), amyotrophic lateral sclerosis (ALS), and Alzheimer’s disease (AD), among others [1–3]. TDP-43 normally localizes to the nucleus, where it plays a critical role in regulating RNA metabolism, including suppressing the inclusion of cryptic exons in mature mRNA transcripts [4]. However, TDP-43 can become depleted from the nucleus upon its mislocalization and aggregation in the cytoplasm, thereby resulting in cryptic exon inclusion [5–8], now considered a pathological hallmark of FTD and ALS [9]. Although cryptic exon inclusion is a well-studied phenomenon of TDP-43 dysfunction, TDP-43 also regulates RNA metabolism by binding GU-rich regions in the 3′ untranslated region (3′ UTR) of its target transcripts [10,11], suggesting a putative role for TDP-43 in regulating the alternative polyadenylation (APA) of these transcripts. In support of this hypothesis, three recent studies, including one of our own in which we used TDP-43-depleted human embryonic stem cells-derived neurons (iNeurons) [12], provided evidence that loss of TDP-43 modulates the 3′ UTR length of critical neuronal genes [12–14], in some cases resulting in transcript instability and a decline in protein expression [12].

The APA of mRNA transcripts modulates their stability and subcellular localization, regulating protein expression [15]. Most mammalian mRNA transcripts have several polyadenylation (PolyA) sites, the varying usage of which can generate different mRNA isoforms with altered 3′ UTR lengths. PolyA sites are flanked by up- and downstream *cis-*regulatory RNA elements that modulate polyA site recognition and processing efficiency [16]. Typically, these elements include a conserved AAUAAA hexamer, or a close variant, upstream of the cleavage site, referred to as the PolyA signal, and GU-rich sequences downstream of the cleavage site that recruit RNA-binding proteins, including TDP-43. Preference for distal PolyA site usage leading to 3′ UTR lengthening is a prominent and conserved feature in neuronal tissues [17]. This predisposes the long isoforms to be bound by miRNAs or RNA-binding proteins, thereby exerting additional post-transcriptional control critical for spatial and temporal gene expression for complex neuronal processes such as synaptic plasticity and neuronal differentiation [18,19]. Therefore, recent work has focused on understanding how RNA-binding proteins such as TDP-43 modulate APA in neurodegenerative diseases.

While traditional RNA sequencing analyses have lacked sensitivity to detect APA events in cellular and tissue models, high-resolution methods have been established to bypass this obstacle [18,20]. For instance, a powerful 3′aTWAS platform was developed to identify previously undetectable APA-linked susceptibility genes that are enriched in pathways related to brain disorders [20]. These and other high-resolution technologies have advanced our ability to detect APA events using in vitro and in vivo model systems [21]. For example, studies employing optimized detection methods in various TDP-43 loss-of-function models have uncovered that nuclear loss of TDP-43 results in widespread alterations in APA events including alternative last exons, 3′ UTR lengthening/shortening, or cryptic intronic PolyA events [12–14]. Moreover, we previously noted that 3′ end sequencing of TDP-43-depleted iNeurons revealed altered PolyA site usage [12]. Of the novel sites we identified, disease-relevant genes such as *TMEM106B*, *SFPQ*, *NEFL*, and *ELP1* demonstrated altered 3′ UTR lengths as well as differential protein expression upon TDP-43 loss of function [12]. Because of altered 3′ UTR lengths, the APA of transcription factors such as *ELK1*, *SIX3*, and *TLX1* led to an increase in their protein expression and function [14]. Moreover, the 3′ UTR lengthening of microtubule affinity regulating kinase 3 led to an increase in neuronal tau S262 phosphorylation [12,13]. Although aberrant APA events caused by TDP-43 depletion have been systematically analyzed and validated in recent studies, the extent to which these APA changes are reflected in the brain of individuals with frontotemporal lobar degeneration with TDP-43 pathology (FTLD-TDP) remain to be elucidated, as do the functional outcomes of these events.

In addition to the aforementioned APA target transcripts, an intriguing finding using TDP-43-depleted iNeurons was the 3′ UTR lengthening of vacuolar protein sorting orthologs 35 and 26B (*VPS35* and *VPS26B*) [12]. VPS35 and VPS26B, together with VPS29, compose the retromer complex, which is essential for modulating endosomal trafficking of cargo throughout the cell [22]. The retromer complex regulates retrograde transport, which is critical for the proper anterograde trafficking of enzymes such as hydrolases and proteases, to the endolysosomal system [22], and is required for the degradation of pathological protein aggregates such as tau and α-synuclein [23]. Additionally, the retromer complex mediates cargo recycling from the endosomes to the cell surface, which is necessary for the recycling of cell surface receptors and other critical molecules, such as glutamate, during synaptic plasticity [24–27]. Mutations in *VPS35* and loss of its protein expression destabilize the retromer complex and promote endolysosomal dysfunction-pathological features commonly observed in AD, Parkinson’s disease, and other neurodegenerative disorders [28,29]. Given its crucial involvement in the trafficking and recycling of proteins involved in synaptic function [22], it is not surprising that retromer dysfunction leads to improper trafficking of well-characterized neurodegenerative disease-associated proteins such as the amyloid precursor protein (APP), and to improper degradation of tau and α-synuclein aggregates [23]. Given that the APA of the retromer components *VPS35* and *VPS26B* is altered upon TDP-43 knockdown [12], and that loss of these proteins is prevalent in neurodegenerative disease [28,29], we set out to explore the ramifications of TDP-43 dysfunction on these retromer components to uncover a potential mechanistic link between TDP-43 loss of function and retromer dysfunction in TDP-43 proteinopathies.

Our present study aimed to (1) investigate whether APA events can be detected in the frontal cortex of FTLD-TDP cases, and to (2) uncover the functional consequences underlying APA changes in FTLD-TDP. To address these aims, we assessed the APA of retromer components *VPS35* and *VPS26B*, and one of the top hits from our prior 3′ end sequencing in iNeurons-the *ELK1* [12,14]. Compared to cognitively normal controls, we observed a significant increase in 3′ UTR lengthening in *VPS35* and *ELK1*, but not *VPS26B*, in FTLD-TDP cases. Strikingly, 3′ UTR lengthening of *VPS35* and *ELK1* was associated with greater frontal cortex cryptic stathmin-2 (*STMN2-CE*) RNA in FTLD-TDP cases. Moreover, *ELK1* APA associated with greater hyperphosphorylated TDP-43 (pTDP-43) and with an earlier age of disease onset, highlighting the importance of APA changes in TDP-43 dysfunction and disease progression. Notably, we observed a significant association between the 3′ UTR lengthening of *VPS35* and decreased VPS35 and VPS29 protein levels, suggesting 3′ UTR lengthening may cause downstream retromer dysfunction. Finally, lower VPS35 protein levels were associated with higher pTDP-43 levels and *STMN2-CE* RNA, further uncovering the functional ramifications of APA alterations in FTLD-TDP. Together, these findings support a role for TDP-43 in modulating APA and implicate TDP-43 loss of function in disrupting the retromer complex in an APA-dependent manner.

## Results

### The 3′ UTRs of *VPS35* and *ELK1* are significantly lengthened in the FTLD-TDP brain

**Fig 1:**
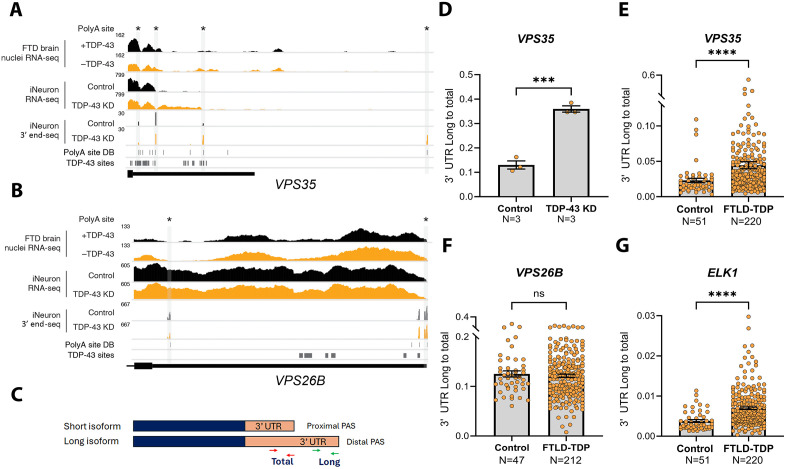
*VPS35* and *ELK1* have longer 3′ UTRs in the frontal cortex of FTLD-TDP cases. **(A, B)** High-resolution APA mapping using 3′ end sequencing in iNeurons revealed that TDP-43 knockdown promotes the usage of novel polyA sites, resulting in 3′ UTR lengthening of *VPS35* (A) and VPS26B (B). For both A and B panels, the first tracks show read coverage from RNA-seq of sorted neuronal nuclei based on TDP-43 levels from the frontal cortex of FTLD-TDP patients [30]. The second tracks show read coverage of RNA-seq from iNeurons treated with either scramble shRNA (control) or with *TARDBP* shRNA to knockdown TDP-43 (TDP-43 KD) [12]. The third tracks show 3′ end-sequencing read coverage from iNeurons treated with either scramble shRNA (control) or *TARDBP* shRNA (TDP-43 KD) [12]. The “PolyA site DB” tracks mark the annotated polyA sites [31], and the “TDP-43 sites” tracks mark TDP-43 binding sites [32]. **(C)** Schematic of the primers used for quantifying the long and total 3′ UTR isoforms using qRT-PCR. **(D)** The ratio of long:total 3′ UTR isoforms of *VPS35* measured using qRT-PCR were significantly enriched in TDP-43 knockdown iNeurons. **(E–G)** Compared to controls, the ratio of long:total 3′ UTR isoforms measured using qRT-PCR were elevated in bulk RNA extracted from the frontal cortex of FTLD-TDP cases for *VPS35* (E) and *ELK1* (G) but not *VPS26B* (F). The number of cases is included in the figure panels. Greater long:total 3′ UTR ratios are indicative of 3′ UTR lengthening. Data are presented as mean ± SEM. Statistical analyses were performed by Mann–Whitney test; ***P < 0.001, ****P < 0.0001. Data used to generate graphs can be found in S1 Data.

To determine whether TDP-43 regulates APA, we previously performed high-resolution 3′ end sequencing in TDP-43-depleted iNeurons. This analysis revealed widespread APA alterations in genes, including genes with established roles in FTLD-TDP/ALS and other neurodegenerative diseases (S1 Table) [12]. Notably, transcripts encoding the retromer proteins, *VPS35* and *VPS26B*, which were not detected using conventional RNA-seq analyses of FTLD-TDP/ALS patient tissues (Fig 1A and 1B, first track) or TDP-43-depleted iNeurons (Fig 1A and 1B, second track), were readily identified when using the more sensitive 3′ end sequencing method (Fig 1A and 1B, third track).

To validate the targets identified by 3′ end sequencing in TDP-43-depleted iNeurons, we designed quantitative real-time PCR (qRT-PCR) primers specific to the long or total 3′ UTR isoforms and calculated the long:total 3′ UTR ratio as a measure of 3′ UTR lengthening (Fig 1C). Using this approach, TDP-43 knockdown iNeurons exhibited a significant increase in *VPS35* 3′ UTR lengthening (Fig 1D), consistent with the 3′ end sequencing results (Fig 1A, third track).

To determine if APA changes observed in iNeurons (S1 Table) were recapitulated in the frontal cortex, we performed qRT-PCR on bulk RNA extracted from the frontal cortex of 220 immunohistologically confirmed FTLD-TDP patients with or without motor neuron disease (MND) and 51 cognitively normal controls (S2 Table). Consistent with the iNeuron data, compared to controls, FTLD-TDP cases showed a significantly higher long:total 3′ UTR ratio for *VPS35* (Fig 1E), but not for *VPS26B* (Fig 1F). Similarly, *ELK1*, one of the top hits from our prior iNeuron study [12], exhibited significant 3′ UTR lengthening in FTLD-TDP frontal cortex (Fig 1G).

Notably, both *VPS35* and *ELK1* displayed substantial shifts in distal polyA site usage (40.8% and 64.4% respectively, S1 Table), whereas *VPS26B* showed a minimal shift (8.6%, S1 Table), indicating that TDP-43-mediated targets with robust distal polyA site changes can be reliably detected by qRT-PCR even in bulk tissue. Based on these findings, we focused on 3′ UTR lengthening of *VPS35* and *ELK1* for further downstream analysis with markers of TDP-43 pathology.

### TDP-43-mediated 3′ UTR lengthening is associated with markers of TDP-43 dysfunction and pathology in the frontal cortex of FTLD-TDP cases

**Fig 2:**
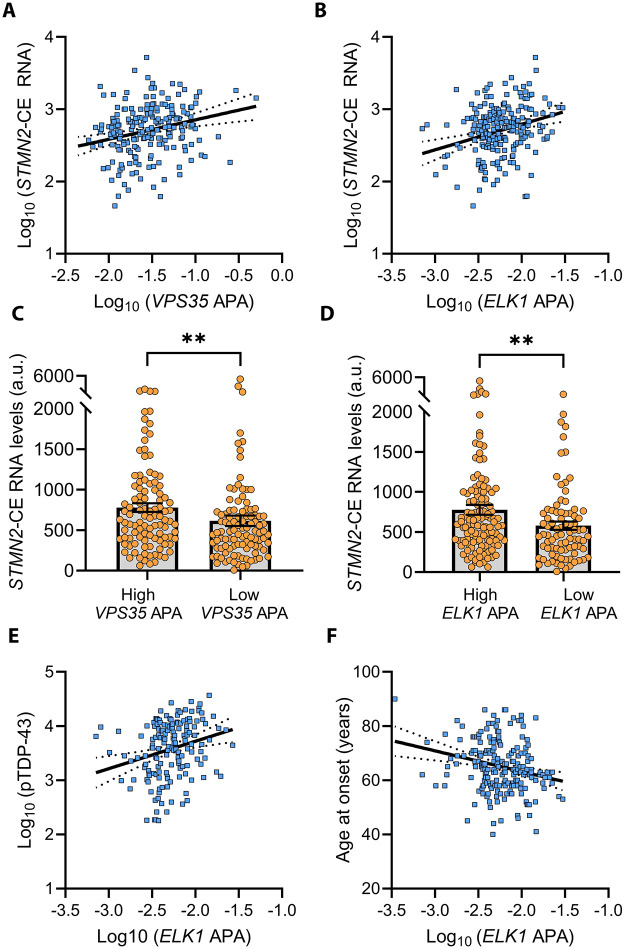
3′ UTR lengthening of *VPS35* and *ELK1* is associated with markers of TDP-43 pathology in the frontal cortex of FTLD-TDP cases. **(A, B)** The ratios of the long to total 3′ UTR isoforms of *VPS35* and *ELK1* in the frontal cortex of FTLD-TDP patients are significantly associated with greater *STMN2-CE* RNA (regression coefficients adjusted for age at death, sex and RIN for *VPS35* and *ELK1*, respectively (*β*: 0.1919, 95% CI: 0.0672 to 0.3165, *P* = 0.0027; and *β*: 0.1827, 95% CI: 0.0764 to 0.2888, *P* = 0.0008; see also S3 Table). **(C, D)** *STMN2-CE* RNA is significantly higher in the High *VPS35* and the High *ELK1* APA groups when compared to their respective Low APA groups (Low APA as the bottom 50%, and High APA as the top 50%, *N* = 104 each). **(E)** *ELK1* APA is significantly associated with higher pTDP-43 levels in unadjusted analysis (*β*: 0.1246, 95% CI: 0.0491 to 0.2001, *P* = 0.0014, see also S5 Table). **(F)** *ELK1* APA is significantly associated with a younger age of disease onset in unadjusted analysis (*β*: −0.0074, 95% CI: −0.0117 to −0.0031, *P* = 0.0008; see also S6 Table) and analysis adjusted for age at death, sex, and RIN (*β*: −0.0077, 95% CI: −0.0121 to −0.0033, *P* = 0.0006; see also S6 Table) in FTLD-TDP patients. Data in C and D are presented as mean ± SEM. Statistical analyses were performed by Mann–Whitney test; **P < 0.01. Data used to generate graphs can be found in S1 Data.

TDP-43 pathology is comprised of TDP-43 nuclear depletion and the accumulation of TDP-43 in the cytoplasm, thereby compromising TDP-43 function [33,34]. TDP-43 dysfunction facilitates the aberrant inclusion of cryptic exons into mature mRNA transcripts, often resulting in their degradation [5–7,35–39]. Therefore, we examined whether cryptic exon inclusion is associated with TDP-43-mediated 3′ UTR lengthening in the frontal cortex of FTLD-TDP cases. We observed that *VPS35* and *ELK1* 3′ UTR lengthening were both significantly associated with increased *STMN2-CE,* a well-characterized TDP-43 RNA target and marker of TDP-43 dysfunction, in unadjusted analysis and in analysis adjusted for age at death, sex, and RNA integrity number (RIN) (Fig 2A and 2B and S3 Table). We also examined associations of two other RNA targets with 3′ UTR lengthening previously identified in TDP-43 knockdown iNeurons: the splicing factor proline and glutamine rich (*SFPQ*), an RNA binding protein associated with the pathogenesis of FTLD-TDP and ALS, and the lysosomal transmembrane protein 106B (*TMEM106B)*, a known genetic risk factor for FTLD-TDP confirmed to exhibit 3′ UTR lengthening in the FTLD-TDP brain [12,40–43]. We observed a significant positive association between the 3′ UTR lengthening of *SFPQ* with *STMN2-CE* RNA, and a trend of 3′ UTR lengthening of *TMEM106B* that associated with *STMN2-CE* RNA (S3 Table). Notably, when stratifying *VPS35* or *ELK1* in FTLD-TDP frontal cortex by APA status (Low APA as the bottom 50%, and High APA as the top 50%), we saw that *STMN2-CE* RNA levels were significantly elevated in the High *VPS35* and High *ELK1* APA groups when compared to their respective Low APA groups (Fig 2C and 2D). Likewise, *STMN2-CE* levels were significantly elevated in the High *SFPQ* and *TMEM106B* APA groups than in their respective Low APA groups, suggesting that APA changes were associated with TDP-43 dysfunction (S1 Fig).

In a similar fashion to the studies above, we examined associations of these same genes with the cryptic form of the hepatoma-derived growth factor-like protein 2 (HDGFL2-CE) produced by TDP-43 dysfunction. In so doing, we noted that only *ELK1* 3′ UTR lengthening was significantly associated with greater HDGFL2-CE protein (S4 Table). Together, these findings strengthen our hypothesis that TDP-43 loss-of-function modulates APA in the FTLD-TDP brain. Finally, to examine whether APA events in the frontal cortex are coordinately dysregulated by TDP-43 pathology in FTLD-TDP, we performed a pairwise Spearman Rank correlation analysis among the APA of *ELK1*, *VPS35*, *SFPQ*, and *TMEM106B* (S2 Fig). While no strong correlations among genes were found, we did observe a moderate correlation between the APA of *VPS35* and *SFPQ* (Spearman *R* = 0.57, *P* < 0.0001), and a weak correlation between the APA of *ELK1* and *SFPQ* (Spearman *R* = 0.16, *P* = 0.017). These findings suggest that TDP-43 dysfunction may only partially contribute to the coordinated dysregulation of some APA events; although it is conceivable that cellular heterogeneity and dynamic APA usage in bulk tissue may mask the coordination among APA events.

We previously linked TDP-43 dysfunction with higher pTDP-43 levels in the frontal cortex or amygdala of FTLD-TDP and AD-TDP cases [7,8,44]. We thus evaluated whether the 3′ UTR lengthening of the above-described transcripts in the frontal cortex of FTLD-TDP patients are associated with higher frontal cortex pTDP-43 levels measured by immunoassay [8,45,46]. Only for *ELK1* APA did we observe a significant association with higher pTDP-43 levels; though this was seen in unadjusted analysis, but not in analyses adjusting for age at death, sex, and RIN (Fig 2E and S5 Table). We did, however, observe significantly higher pTDP-43 levels in the High *ELK1* APA group compared to the Low *ELK1* APA group (S3 Fig). When examining associations of *VPS35* APA with pTDP-43 levels, we noted a nominally significant positive association of *VPS35* APA with pTDP-43 in unadjusted analysis (S4 Fig and S5 Table) but saw no difference in pTDP-43 levels between the High *VPS35* and Low *VPS35* APA groups (S3 Fig). These findings suggest that the presence of pTDP-43 may influence APA dysregulation in a transcript-dependent manner.

Previously, we established that *STMN2-CE* RNA, a marker of TDP-43 dysfunction, is associated with an earlier age at disease onset [8]. Because APA changes are also associated with TDP-43 dysfunction, we hypothesized that APA alterations may also be associated with FTLD-TDP clinical characteristics. We found that 3′ UTR lengthening of *ELK1*, but not of *VPS35*, *SFPQ*, or *TMEM106B*, was significantly associated with an earlier age at disease onset in both unadjusted analysis and in analysis adjusted for sex, RIN, and the presence of MND (Fig 2F and S6 Table). In analysis adjusted for age at onset, sex, RIN, and the presence of MND, we also observed that 3′ UTR lengthening of *ELK1* was associated with a shorter disease duration (i.e., the time between symptom onset to death) (S6 Table). These data suggest that TDP-43-mediated 3′ UTR lengthening events occur early in disease pathogenesis and hasten disease progression.

### TDP-43-mediated 3′ UTR lengthening of *VPS35* correlates with retromer dysfunction in the frontal cortex of FTLD-TDP cases

**Fig 3:**
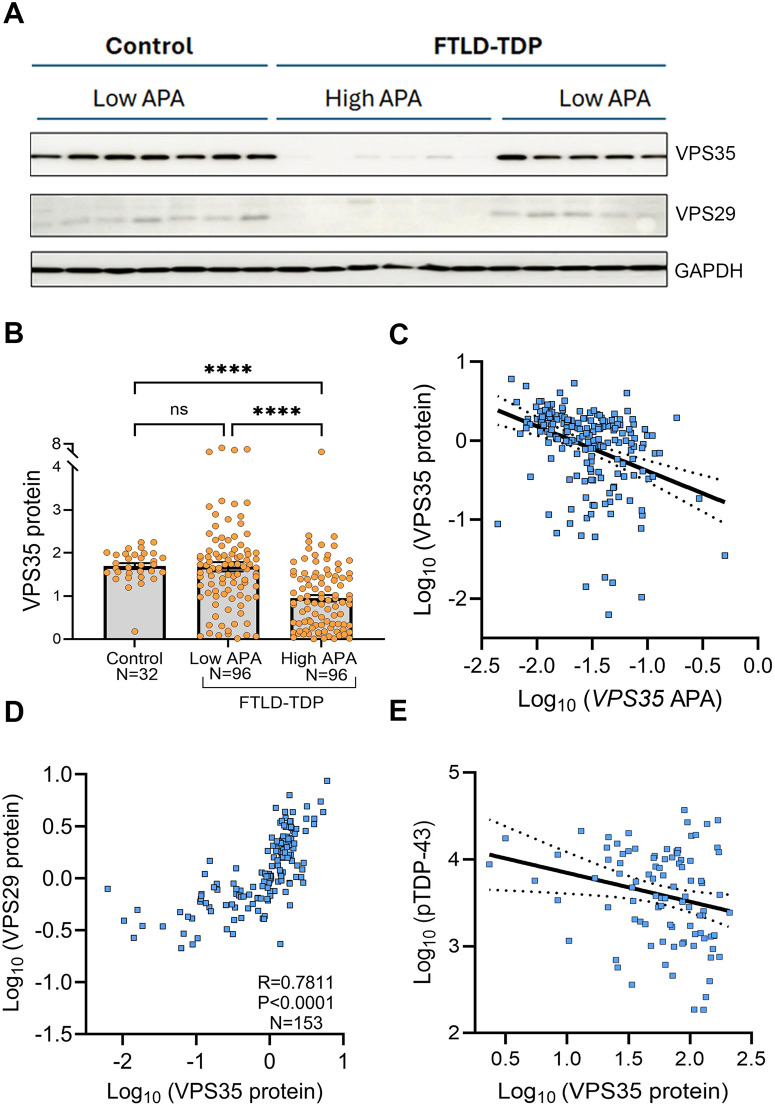
3′ UTR lengthening of *VPS35* modulates retromer components. **(A)** Representative western blot image of VPS35 and VPS29 protein levels from the RIPA soluble fraction extracted from the frontal cortex of healthy controls and from patients with FTLD-TDP; the latter group was dichotomized using median 3′ UTR lengthening as the cutoff. GAPDH: Loading control. **(B)** Frontal cortex VPS35 protein was significantly lower in the High *VPS35* APA FTLD-TDP cases compared to the FTLD-TDP cases with Low *VPS35* APA or to the healthy controls. Data are presented as mean ± SEM. Statistical analyses were performed using a non-parametric, Kruskal–Wallis test, **** *P* < 0.0001. **(C)** 3′ UTR lengthening of *VPS35* was significantly associated with lower VPS35 protein in the frontal cortex of FTLD-TDP cases (regression coefficient, adjusted for age at death, sex, and RIN, *β*: −0.2109, 95% CI: (−0.3092 to −0.1127), *P* < 0.0001, S7 Table). **(D)** Higher VPS35 protein levels correlated with higher VPS29 protein levels in the frontal cortex of FTLD-TDP cases; data shown are from Spearman rank correlation (*R* = 0.7811, *P* < 0.0001). **(E)** Higher pTDP-43 levels in FTLD-TDP were negatively associated with VPS35 protein levels in the frontal cortex of FTLD-TDP cases; data shown are from linear regression models (regression coefficient adjusted for age at death, sex, and presence of MND, *β*: −0.0026, 95% CI: (−0.0047 to −0.0005), *P* = 0.0147, S8 Table). *P*-values and sample sizes are indicated in the graphs. Simple linear regression fit is indicated with a solid black line, and the 95% confidence bands of the fit line are indicated by dotted lines. Data used to generate graphs can be found in S1 Data.

Retromer dysfunction caused by the mutation or reduction of retromer component proteins are commonly observed in neurodegenerative diseases including AD and Parkinson’s disease [47–50]. Recently, a proteomic study found that VPS35 protein levels were decreased in TDP-43 knockdown iNeurons compared to control iNeurons [51]. Consistent with this finding, we observed that TDP-43 knockdown in iNeurons, which causes *VPS35* 3′ UTR lengthening, results in decreased VPS35 protein levels (S5 Fig). To test whether TDP-43-mediated 3′ UTR lengthening of *VPS35* alters its protein levels, we measured VPS35 protein in the frontal cortex of healthy controls and FTLD-TDP patients by western blot and stratified the FTLD-TDP group by APA status (Low APA as the bottom 50%, and High APA as the top 50%). Remarkably, VPS35 protein expression was significantly lower in FTLD-TDP cases with High APA compared to FTLD-TDP cases with Low APA or to the healthy controls (Figs 3A and 3B and S1 Raw Images). We also found that *VPS35* 3′ UTR lengthening was associated with lower VPS35 protein in unadjusted analysis and analysis adjusted for age at death, sex, and RIN (Fig 3C and S7 Table).

In the multimeric retromer complex, loss of one subunit can alter the levels of other retromer components or its binding partners [52]. Therefore, we examined whether 3′ UTR lengthening of *VPS35* disrupts the VPS29 retromer subunit. Of interest, higher *VPS35* APA was significantly associated with lower VPS29 protein levels in both unadjusted analysis and analysis adjusted for age at death, sex, and RIN (S7 Table). Consistent with data from previous studies, we observed a striking correlation between VPS29 and VPS35 protein levels (Spearman *R* = 0.7811, *P* < 0.0001, Fig 3D). Loss of VPS35 protein in FTLD-TDP was also found to associate with increased pTDP-43 levels (Fig 3E and S8 Table) and with elevated *STMN2-CE* RNA (S9 Table). In aggregate, these findings provide evidence that TDP-43 dysfunction modulates the APA of retromer component *VPS35*, reducing the expression of VPS35 and VPS29, thereby uncovering a potential mechanistic link between TDP-43 loss of function, APA, and retromer dysfunction in TDP-43 proteinopathies.

## Discussion

Here, we explored whether previously identified APA targets, such as *VPS35* and *ELK1*, demonstrate conserved 3′ UTR lengthening in the frontal cortex of a large cohort of patients with FTLD-TDP. In so doing, we uncovered associations of TDP-43-mediated APA changes with markers of TDP-43 dysfunction and clinical characteristics. In FTLD-TDP cases, we observed 3′ UTR lengthening of *VPS35* and *ELK1*, but not of *VPS26B*. Moreover, *VPS35*, *ELK1*, and *SFPQ* 3′ UTR lengthening was found to associate with elevated *STMN2-CE* RNA. *ELK1* 3′ UTR lengthening was also associated with higher HDGFL2-CE and pTDP-43 protein levels, and with an earlier age at disease onset. In line with these findings, when stratifying *VPS35*, *ELK1*, *SFPQ*, and *TMEM106B* by high or low APA status, we observed that *STMN2-CE* RNA was elevated in the high APA groups. These findings support APA alterations as a critical consequence of TDP-43 dysfunction in FTLD-TDP.

We also investigated how TDP-43 dysfunction in FTLD-TDP influences the APA of *VPS35,* a key component of the retromer complex, and found that TDP-43 loss of function promotes 3′ UTR lengthening of *VPS35* thereby decreasing VPS35 and VPS29 protein levels. Our data thus connect TDP-43 loss of function and retromer dysfunction in FTLD-TDP. Moreover, we uncovered that VPS35 protein levels are inversely associated with pTDP-43 and *STMN2-CE* RNA levels. Although multiple mechanisms contribute to retromer dysfunction, two key retromer components—VPS35 and VPS26B—play central roles in modulating retromer integrity, both of which are commonly suppressed in the AD brain [53]. It also bears mentioning that, in a VPS35 knockout mouse model, loss of Vps35 has been associated with neurodegenerative pathology, resulting in pTDP-43 and p62 accumulation, suggesting that loss of VPS35 protein exacerbates TDP-43 pathology [54]. Because VPS35 is critical for stabilizing the retromer complex, its depletion can reduce other retromer components, such as VPS29, ultimately impairing retrograde and downstream anterograde transport of proteases essential to lysosomal function [22]. Given the importance of the retromer in modulating protein trafficking, and its implications in neurodegenerative disease, pharmacological interventions have been designed to help stabilize retromer components and maintain protein trafficking, demonstrating the potential for modulating the processing and clearance of APP and pTau, two AD-related proteins [55,56]. TDP-43-mediated 3′ UTR lengthening of *VPS35* may thus connect TDP-43 pathology to downstream retromer dysfunction, as observed in TDP-43 proteinopathies.

**Fig 4:**
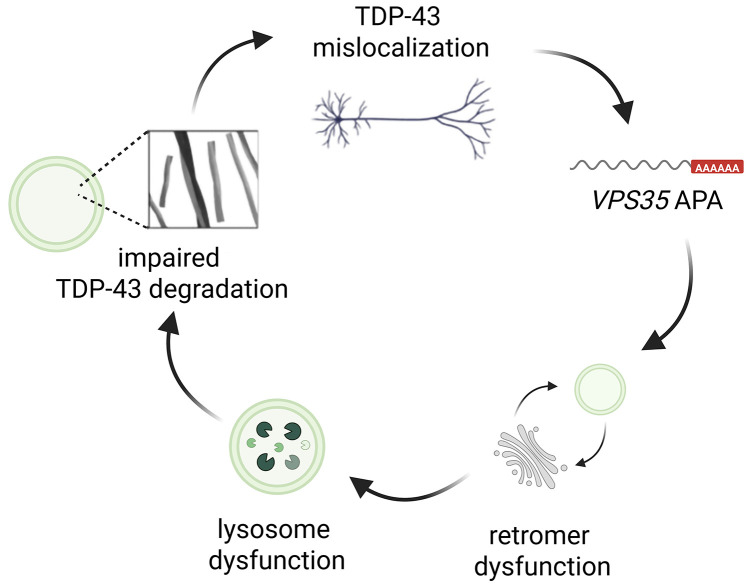
The complex interplay between TDP-43 loss of function, APA changes, and retromer/endolysosomal dysfunction in FTLD-TDP. Our data support a model in which TDP-43 loss of function triggers APA changes in key transcripts, including the retromer core component *VPS35*, resulting in reduced VPS35 and VPS29 protein levels and, subsequently, retromer dysfunction. In turn, endolysosomal dysfunction diminishes TDP-43 degradation, exacerbating TDP-43 mislocalization and loss of function in TDP-43 proteinopathies. Created in BioRender. Maheswari Jawahar, V. (2025) https://BioRender.com/b9nif6c.

These findings support a mechanistic model in which TDP-43 loss of function in FTLD-TDP triggers TDP-43-mediated APA, initiating retromer dysfunction and consequently endolysosomal failure thereby promoting the accumulation of disease-relevant proteins such as pathological TDP-43, which in turn exacerbates TDP-43 dysfunction (Fig 4). Endolysosomal impairment is widely implicated in TDP-43 proteinopathies, including AD, FTD/ALS, and Parkinson’s disease [57–59], and our prior studies established a role for endolysosomal dysfunction in inducing TDP-43 proteinopathy in FTD/ALS [60]. The present study extends this framework by positioning TDP-43 dysfunction-induced APA dysregulation as an upstream event precipitating retromer destabilization and endolysosomal dysfunction in FTLD-TDP.

Single-cell proteomics of postmortem human spinal motor neurons have revealed that retromer deficiency accompanies TDP-43 neuronal cytoplasmic aggregates (TDP-43+ NCI) [61], and that retromer-targeted pharmacological chaperones confer neuroprotection in ALS [62]. However, these findings were derived from a combined cohort of patients with TDP-43+ NCI and controls, and their significance was diminished when controls were excluded [61]. Our analysis examining associations of TDP-43 dysfunction and 3′ UTR lengthening events were conducted exclusively in FTLD-TDP patient tissues, revealing a more robust association between TDP-43 pathology and retromer dysfunction. Moreover, previous findings by our group and others support a continuous loop whereby endolysosomal dysfunction exacerbates TDP-43 pathology [58,60,63,64], further accelerating disease progression. Consistent with this notion, we recently uncovered that loss of the lysosomal transport complex BORC increases the half-life of TDP-43, exacerbating resulting pathology [64]. Building on this concept, data from our present study suggests that TDP-43 loss-of-function acts upstream of endolysosomal dysfunction by modulating APA changes, and that targeting these alternative functions of TDP-43 may offer a therapeutic strategy to mitigate disease pathology and progression. Together, these findings support a link between TDP-43 dysfunction, APA changes, retromer deficiency, and endolysosomal dysfunction. The addition of APA to this mechanistic axis is essential for understanding how TDP-43 loss of function drives downstream molecular dysfunction in TDP-43 proteinopathies.

In contrast to previous studies utilizing sorted TDP-43-negative nuclei [12–14], here we targeted bulk postmortem frontal cortex tissue for our APA analysis. However, inherent cellular heterogeneity of the human brain and the complexity of FTD/ALS pathophysiology may dilute these effects. Future studies employing single-cell sequencing, proteomics, and RNA-FISH techniques optimized on human postmortem samples should allow a thorough exploration of how APA changes occur in cellular subtypes, a significant step in untangling the molecular consequences of TDP-43 dysfunction. Moreover, the widespread ramifications of TDP-43-mediated APA in other neurodegenerative diseases and brain regions is yet to be explored and should be a focus of future investigation. Overall, our study provides compelling evidence that TDP-43 dysfunction exacerbates retromer deficiency by altering the APA of *VPS35*, thereby contributing to endolysosomal impairment. These findings highlight APA–retromer regulation as a potential therapeutic target for mitigating downstream effects of TDP-43 dysfunction in neurodegenerative diseases.

## Materials and methods

### Ethics statement

Human postmortem brain tissues from FTLD-TDP patients were provided by the Mayo Clinic Florida Brain Bank, with neuropathological diagnosis determined by a single neuropathologist (D.W.D.). Written informed consent was obtained from all subjects (or their legal next of kin if they were unable to give written consent) prior to study entry, and all protocols were approved by the Mayo Clinic Institutional Review Board.

### Study cohort characteristics

Our study cohort included 271 postmortem tissue samples: 51 healthy controls and 220 FTLD-TDP cases. Patient characteristics of this cohort are as described in S2 Table. Average RIN values were 9.45 and 9.12 for healthy controls and FTLD-TDP, respectively. Among the FTLD-TDP cohort, 25.9% of cases were also neuropathologically confirmed MND cases.

### Visualization of read coverage

3′ end sequencing data generated from our prior study using iNeurons are available at GEO: GSE252892 [12]. The following publicly available GEO data were downloaded from GSE126542 and prepared for visualization using IGV. We gathered annotated polyA sites and TDP-43 binding sites from previous references [31,32].

### RNA extraction and cDNA synthesis in postmortem tissues

RNA was extracted from the frontal cortex of healthy cases and cases with FTLD-TDP following the manufacturer’s protocol using the RNeasy Plus Mini Kit (Qiagen) as previously described [65]. The RNA concentration was quantified using a NanoDrop (Thermo Fisher) and the RIN was measured by the Agilent 2100 Bioanalyzer (Agilent Technologies). Five hundred nanograms of total RNA was transcribed into complementary DNA (cDNA) according to the manufacturer’s instructions using the High-Capacity cDNA Transcription Kit (Applied Biosystems).

### TDP-43 knockdown in iNeurons

Stem cell maintenance and differentiation into iNeurons were followed as previously described [12]. In brief, human embryonic stem cells (hESCs; H1) were cultured in mTeSR1 Plus medium (StemCell Technologies, 100-0276) on Matrigel-coated surfaces (Corning, 354230). Cells were fed every other day and passaged every 4–7 days using ReLeSR (StemCell Technologies, 100-0483) following the manufacturer’s protocol. Neuronal differentiation was induced by overexpressing NGN2. On day 3 of differentiation, the cells were dissociated and re-plated on Matrigel-coated plates in Neurobasal Medium (Thermo Fisher, 21103049) supplemented with the neurotrophic factors BDNF and GDNF (R&D Systems). After 7 days of differentiation, iNeurons were transduced with lentivirus expressing either scramble shRNA or TDP-43 shRNA and cultured for 12 days, before harvesting for further analysis.

### qRT-PCR analysis of long and total 3′ UTR isoforms

To quantify the expression of long and total 3′ UTR isoforms in iNeurons or frontal cortices of control or FTLD-TDP cases, triplicates of the cDNA samples were used for amplifying the target genes using SYBR GreenER qPCR SuperMix (Invitrogen) on a QuantStudio 7 Flex Real-Time PCR System (Applied Biosystems). Relative quantification of the long and total 3′ UTR isoforms were determined using the ΔΔCt method and normalized with the geometric mean of two endogenous controls, GAPDH and RPLP0. The primers used were as follows: *VPS35* total 3′ UTR forward 5′-CCATGTACATCCAGTGAGG, reverse: 5′-GGTCCTGAAGGTGAGTGTC; VPS35 long 3′ UTR forward 5′-GAGAAATCTGTGCAGGAGACC, reverse 5′-CCTAAGGCAAGTCACTACTC; *ELK1* total 3′ UTR forward 5′-GGACAGTGCTACACTCGTC, reverse: 5′-TACAGGAACAATTCCCCATTCTC; *ELK1* long 3′ UTR forward 5′-ACTTTACTGCCCATAAAACAAGTG, reverse 5′-GAAAGATAGGAAGGCAATGCC; GAPDH forward: 5′-GTTCGACAGTCAGCCGCATC, Reverse: 5′-GGAATTTGCCATGGGTGGA; and RPLP0 forward: 5′-TCTACAACCCTGAAGTGCTTGAT, Reverse: 5′-CAATCTGCAGACAGACACTGG. The ratio of long to total 3′ UTR isoforms was used as a measure 3′ UTR lengthening.

### NanoString analyses for measuring *STMN2-CE* RNA levels

Two hundred fifty nanograms of RNA with a RIN value greater than 7.0 were used to measure *STMN2-CE* RNA using the NanoString PlexSet platform, according to the manufacturer’s instructions. Transcript-level abundance was analyzed using nSolver 4.0 software (NanoString Technologies) and normalized to the endogenous control *HTRP1,* which was unaltered across disease subtypes [8].

### HDGFL2-CE MSD assay

The HDGFL2-CE MSD assay was performed following the previously described protocol with a few modifications [45,51]. To quantify HDGFL2-CE in the RIPA soluble fraction of the frontal cortices of the control or FTLD-TDP cases, we employed MSD GOLD 96-well Small Spot Streptavidin plates. Wells were coated overnight at 4 °C with 30 µL capture antibody (4 µg/mL). The plates were then washed once with 1× MSD wash buffer and blocked with 150 µL of 0.3% MSD Blocker A diluted in 1× TBST (Tris-buffered saline with 0.1% Tween-20) while shaking at 600 rpm for 1 hour at room temperature. Next, samples prepared in MSD Diluent 100 were tested in duplicate using 32.5 μg of protein per well. The plates were then shaken at 600 rpm for 10 min at room temperature and incubated overnight at 4 °C, without shaking. The following day, the plates were washed with 1× MSD buffer three times, each wash using 150 µL per well, and the sulfo-tagged detection antibody (4 µg/mL) was added for a 2-hour incubation at room temperature with shaking at 600 rpm. After washing, 150 µL of MSD GOLD Read Buffer A was added to each well and the plate was placed into the MSD QUICKPLEX SQ120 instrument, where the electrochemiluminescence signal was measured (in arbitrary units, A.U.) corresponding to the intensity of emitted light, which reflects the abundance of HDGFL2-CE protein in each sample. Background signals obtained from wells containing MSD diluent alone were subtracted from the sample measurements.

### pTDP-43 immunoassay

The pTDP-43 immunoassay was performed as previously described [8,65]. In brief, 50 mg postmortem tissue from the frontal cortex of FTLD-TDP patients or healthy controls were homogenized in cold RIPA buffer (25 mM Tris-HCl, pH 7.6, 150 mM NaCl, 1% sodium deoxycholate, 1% Nonidet P-40, 0.1% sodium dodecyl sulfate, and protease and phosphatase inhibitors) and sonicated on ice. Homogenates were then centrifuged at 100,000*g* for 30 min at 4 °C. After centrifugation, the supernatant was collected separately, and the pellet was resuspended in RIPA buffer, sonicated, and centrifuged again to reduce the carryover soluble protein. The RIPA-insoluble pellet was extracted using 7 M urea buffer and then sonicated and centrifuged at 100,000*g* for 30 min at 22 °C. The Bradford assay was used to measure the protein concentration of the urea-soluble supernatant. The MSD immunoassay was used to determine pTDP-43 levels. In brief, 35 mg of the urea-soluble fraction was diluted in TBS and tested in duplicates. We used a mouse monoclonal antibody to capture phosphorylated TDP-43 at serines 409/410 (1:500, Cosmo Bio USA) and a sulfo-tagged rabbit polyclonal antibody to detect C-terminal TDP-43 (2 μg/mL, Proteintech). Response values corresponding to the intensity of emitted light upon electrochemical stimulation of the assay plate were acquired using the MSD QUICKPLEX SQ120.

### RIPA-soluble fraction extraction and immunoblotting

To quantify soluble VPS35 protein, we generated a RIPA-soluble fraction by homogenizing postmortem frontal cortex samples in 5 volumes (w/v) of ice-cold RIPA buffer (25 mM Tris-HCl pH 7.5, 150 mM NaCl, 1% Nonidet P-40, 1% sodium deoxycholate, 0.1% SDS, with protease and phosphatase inhibitors) as previously described [41]. Homogenates were sonicated (1 s on/1 s off for 10 s) and centrifuged at 100,000*g* for 30 min at 4 °C. The supernatant collected is referred to hereafter as the RIPA-soluble fraction. Twenty μg of the RIPA soluble fraction was diluted with 2×  SDS gel loading buffer at a 1:1 ratio (v/v) and then heated at 95 °C for 5 min. Samples were then loaded into 10- or 20-well 4%–20% Tris-glycine gels (Novex), and after running gels, were transferred to PVDF membranes. The membranes were blocked with 5% nonfat dry milk in TBS plus 0.1% Triton X (TBST) for 1 hour, and then incubated with primary antibodies for VPS35 (1:1,000, Bethyl laboratories), VPS29 (1:1,000, Santa cruz Biotechnology), and GAPDH (1:1,000, Meridian Bioscience) overnight while rocking at 4 °C. The next day, membranes were washed in TBST and incubated with donkey anti-rabbit or anti-mouse IgG antibodies conjugated to horseradish peroxidase (1:5,000; Jackson ImmunoResearch) for 1 hour. Protein expression was visualized by enhanced chemiluminescence treatment and exposure to film. Band intensities were quantified using ImageJ [66].

### Sarkosyl-insoluble fraction extraction and immunoblotting

To quantify insoluble VPS35 protein, we generated a sarkosyl-insoluble P3 fraction. In brief, human postmortem frontal cortex tissue was homogenized in a cold buffer at a 1:5 weight-to-volume ratio. The buffer contained 10 mM Tris-HCl (pH 7.4), 80 mM NaCl, 1 mM MgCl_2_, 1 mM EGTA, 0.1 mM EDTA, 1 mM PMSF, 1 mM dithiothreitol, and a cocktail of protease and phosphatase inhibitors. After homogenization, 400 µL of the homogenate (equivalent to 80 mg of brain tissue) was subjected to ultracentrifugation at 150,000 *g* for 40 min at 4 °C using a TLA110 rotor at 60,000 rpm. The resulting pellet was reconstituted in an equal volume of cold buffer containing 10 mM Tris (pH 7.4), 0.85 M NaCl, 10% sucrose, and 1 mM EGTA, followed by centrifugation at 14,000 *g* for 10 min at 16 °C. The supernatant was then treated with 1% Sarkosyl and agitated continuously at room temperature for 1 hour. Following incubation, samples were again ultracentrifuged at 150,000 *g* for 40 min at 4 °C using the same rotor. The Sarkosyl-insoluble pellet (P3 fraction) was resuspended in 50 µL of TE buffer (10 mM Tris-HCl, 1 mM EDTA), and 10 µL of the sample was used for VPS35 (insoluble) western blot analysis.

### Quantification and statistical analysis

All statistical analyses, apart from S8 and S9 Tables, were performed using GraphPad Prism 10 (GraphPad Software). To compare the long to total 3′ UTR RNA levels between healthy and FTLD-TDP cases, the non-parametric Mann–Whitney test was used. Comparisons of long to total 3′ UTR isoforms in the frontal cortex with the markers of TDP-43 loss of function such as *STMN2-CE* RNA, HDGFL2-CE protein, and pTDP-43 levels were performed using single-variable (i.e., unadjusted) and multivariable linear regression models. The multivariable model assessing the effect of the APA changes with *STMN2-CE* RNA, HDGFL2-CE protein, or pTDP-43 levels were adjusted for age at death, sex, and RIN. The multivariable model assessing the effect of the APA changes with age at onset were adjusted for sex, RIN, and the presence of MND. The multivariable model assessing the effect of the APA changes and disease duration were adjusted for sex, RIN, age at onset, and the presence of MND. Regression coefficients (referred to as *β*) and 95% CIs were estimated from the linear regression model. For S8 and S9 Tables, simple linear regressions were performed using R version 4.4.1 (2024-06-14) [67]. The statistical test and sample sizes used for each analysis are indicated in the figure legends.

## Supporting information

S1 Fig High *SFPQ* and *TMEM106B* APA in FTLD-TDP frontal cortex show elevated *STMN2-CE* RNA. High *SFPQ* APA (**A**) and *TMEM106B* APA (**B**) have significantly higher *STMN2-CE* RNA when compared to their respective Low APA group (Low APA as the bottom 50%, and High APA as the top 50%, *N* = 104 each). Data are presented as mean ± SEM. Statistical analyses were performed by Mann–Whitney test: *P < 0.05. Data used to generate graphs can be found in S1 Data.
(TIF)

S2 Fig TDP-43-dependent APA events exhibit mild to moderate coordination. Heatmap demonstrating the pairwise APA concordance among the four targets: *ELK1*, *VPS35*, *SFPQ*, and *TMEM106B* APA using Spearman Rank correlation. A statistically significant positive association between the APA of *VPS35* and *SFPQ* (Spearman *R* = 0.57, *P* < 0.0001), and a weak, but statistically significant positive association between the APA of *ELK1* and *SFPQ* (Spearman *R* = 0.16, *P* = 0.017) were observed. Data used to generate graphs can be found in S1 Data.
(TIF)

S3 Fig pTDP-43 levels are elevated in FTLD-TDP cases with high *ELK1* APA compared to low *ELK1* APA cases. **(A)** When stratifying *ELK1* APA by high or low APA status (Low APA as the bottom 50%, and High APA as the top 50%, *N* = 80 each), we observed that pTDP-43 was elevated in the high APA group compared to the low *ELK1* APA. (**B**) No difference in pTDP-43 levels was observed between the high and low *VPS35* APA groups. Data are presented as mean ± SEM. Statistical analyses were performed by Mann–Whitney test: ***P < 0.001, ns, not significant. Data used to generate graphs can be found in S1 Data.
(TIF)

S4 Fig *VPS35* APA exhibits a nominally significant association with frontal cortex pTDP-43 levels in unadjusted analysis. We observed a nominally significant, positive association of *VPS35* APA with pTDP-43 levels (*β*: 0.1084, 95% CI: 0.0064 to 0.2103, *P* = 0.0373, S5 Table). No significant association was observed in analysis adjusting for age at death, sex, and RIN (*β*:0.1044, 95% CI:0.0014 to 0.2103, *P* = 0.0533, S5 Table). The simple linear regression fit is indicated by a solid black line, and the 95% confidence intervals (CI) are indicated by the dotted lines. Data used to generate graphs can be found in S1 Data.
(TIF)

S5 Fig Loss of TDP-43 in iNeurons shows a reduction in VPS35 protein levels. **(A)** Western blot image of VPS35 protein levels in iNeurons with TDP-43 knockdown (Loading control: α-tubulin). **(B)** Quantitation of the VPS35 protein levels.
(TIF)

S1 Raw Images Unedited western blot data. Raw data for Fig 3A. VPS35 and VPS29 protein levels were significantly decreased in FTLD-TDP patients with High *VPS35* APA compared to FTLD-TDP patients with Low *VPS35* APA and to healthy controls. RIPA soluble protein fractions were extracted from the frontal cortex of the healthy controls and FTLD-TDP patients. GAPDH: Loading control. Raw data for S5 Fig. VPS35 protein levels in iNeurons with TDP-43 knockdown.
(TIF)

S1 Table Validation of targets identified by 3′ end sequencing in TDP-43 knockdown iNeurons in the frontal cortex of FTLD-TDP cases using qRT-PCR. The delta_distal_PAS_usage and padj from 3**′**  end sequencing in TDP-43 knockdown iNeurons were described previously in Zeng and colleagues [12]. APA changes measured using 3′ end sequencing were considered significant if the adjusted *P*-value was <0.05.
(DOCX)

S2 Table Patient characteristics in postmortem cohorts of healthy controls and FTLD-TDP cases. The sample mean (minimum, maximum) is given for continuous variables, and number (%) of cases is provided for categorical variables. Information was unavailable for a subset of FTLD-TDP patients regarding age at onset (*N* = 20), disease duration (*N* = 19), and age at death (*N* = 2).
(DOCX)

S3 Table TDP-43-mediated 3′ UTR lengthening is associated with *STMN2-CE* RNA in the frontal cortex of FTLD-TDP cases. CI, confidence interval; Regression coefficients, 95% CIs, and *P*-values result from unadjusted linear regression models or linear regression models adjusted for sex, RIN, and age at death where APA and *STMN2-CE* RNA levels were considered on the base 10 logarithmic scale. *STMN2-CE* RNA levels were measured on a subset of FTLD-TDP cases, *N* = 206. *P*-values <**0.0125** are considered statistically significant after correcting for multiple testing. Significance is denoted by bolded text.
(DOCX)

S4 Table TDP-43-mediated 3′ UTR lengthening of *ELK1* is associated with HDGFL2-CE protein in the frontal cortex of FTLD-TDP cases. CI, confidence interval; Regression coefficients, 95% CIs, and *P*-values result from unadjusted linear regression models or linear regression models adjusted for sex, RIN, and age at death where APA and HDGFL2-CE protein levels were considered on the base 10 logarithmic scale. *P*-values **<0.0125** are considered statistically significant after correcting for multiple testing. Significance is denoted by bolded text.
(DOCX)

S5 Table TDP-43-mediated 3′ UTR lengthening of *ELK1* is associated with pTDP-43 burden in the frontal cortex of FTLD-TDP cases. CI, confidence interval; Regression coefficients, 95% CIs, and *P*-values result from unadjusted linear regression models or linear regression models adjusted for sex, RIN, and age at death where APA levels were considered on the base 10 logarithmic scale. pTDP-43 levels were measured on a subset of FTLD-TDP cases, *N* = 160. *P*-values **<0.0125** are considered statistically significant after correcting for multiple testing. Significance is denoted by bolded text.
(DOCX)

S6 Table TDP-43-mediated 3′ UTR lengthening of *ELK1* is associated with clinical characteristics in FTLD-TDP patients. CI, confidence interval; Regression coefficients, 95% CIs, and *P*-values result from unadjusted and adjusted linear regression models where APA levels were considered on the base 10 logarithmic scale. *P*-values **<0.0125** are considered statistically significant after correcting for multiple testing. Multivariable analysis associations of APA with age at onset was corrected for sex, RIN, and presence of MND. Multivariable analysis associations of APA with disease duration (time between age of onset and time) were corrected for sex, RIN, age at onset, and presence of MND. Significance is denoted by bolded text.
(DOCX)

S7 Table *VPS35* 3′ UTR lengthening is associated with VPS35 and VPS29 protein levels in the frontal cortex of FTLD-TDP cases. CI, confidence interval; Regression coefficients, 95% CIs, and P-values result from unadjusted linear regression models or linear regression models adjusted for sex, RIN, and age at death where *VPS35* RNA and protein levels were considered on the base 10 logarithmic scale. Sample size of *N* = 192 for VPS35 protein levels and *N* = 153 for VPS29 protein levels were used depending on sample availability. *P*-values **<0.025** are considered statistically significant after correcting for multiple testing. Significance is denoted by bolded text.
(DOCX)

S8 Table VPS35 protein is associated with pTDP-43 burden in the frontal cortex of FTLD-TDP cases. *β*, regression coefficient; CI, confidence interval; AIC, Akaike Information Criterion. *β*, 95% CIs, AICs, and *P*-values result from unadjusted linear regression models or linear regression models adjusted for either age at death and sex or age at death, sex, and presence of MND where pTDP-43 levels were considered on the base 10 logarithmic scale. Beta coefficients can be interpreted as the change in log_10_(pTDP-43) levels given a one-unit increase in VPS35 protein levels in the insoluble fraction. Lower AIC scores indicate that a model produces a better fit to the data. After applying Bonferroni correction, adjusted *p*-values **<0.0167** were determined to be significant. Significance is denoted by bolded text.
(DOCX)

S9 Table VPS35 protein is associated with *STMN2-CE* RNA in the frontal cortex of FTLD-TDP cases. *β*, regression coefficient; CI, confidence interval; AIC, Akaike Information Criterion. *β*, 95% CIs, AICs, and *P*-values result from unadjusted linear regression models or linear regression models adjusted for either age at death and sex or age at death, sex, and presence of MND. VPS35 protein shows a significant inverse association with *STMN2-CE* RNA levels in the FTLD-TDP postmortem brain. Significance is denoted by bolded text.
(DOCX)

S1 Data Data used to generate graphs in Figs 1, 2, 3, S1, S2, S3, S4, and S5 and S2–S9 Tables. (XLSX)

## Abbreviations

AD: Alzheimer’s disease
ALS: Amyotrophic lateral sclerosis
APA: Alternative polyadenylation
APP: Amyloid precursor protein
ELK1: ETS transcription factor
FTD: Frontotemporal dementia
FTLD-TDP: Frontotemporal lobar degeneration with TDP-43 pathology
HDGFL2-CE: Hepatoma-derived growth factor-like protein 2-cryptic exon isoform
MND: Motor neuron disease
PolyA: polyadenylation
pTDP-43: hyperphosphorylated TDP-43
qRT-PCR: quantitative real-time PCR
RIN: RNA integrity number
SFPQ: Splicing factor proline and glutamine rich
TDP-43: TAR DNA-binding protein 43
VPS26B: Vacuolar protein sorting 26B
VPS35: Vacuolar protein sorting 35
