# Supplementary figures and images for: TDP-43-mediated alternative polyadenylation is associated with a reduction in VPS35 and VPS29 expression in frontotemporal dementia
Source: PLoS Biol. 2026 Jan 5;24(1):e3003573. doi: 10.1371/journal.pbio.3003573 (PMC12768243; doi:10.1371/journal.pbio.3003573)

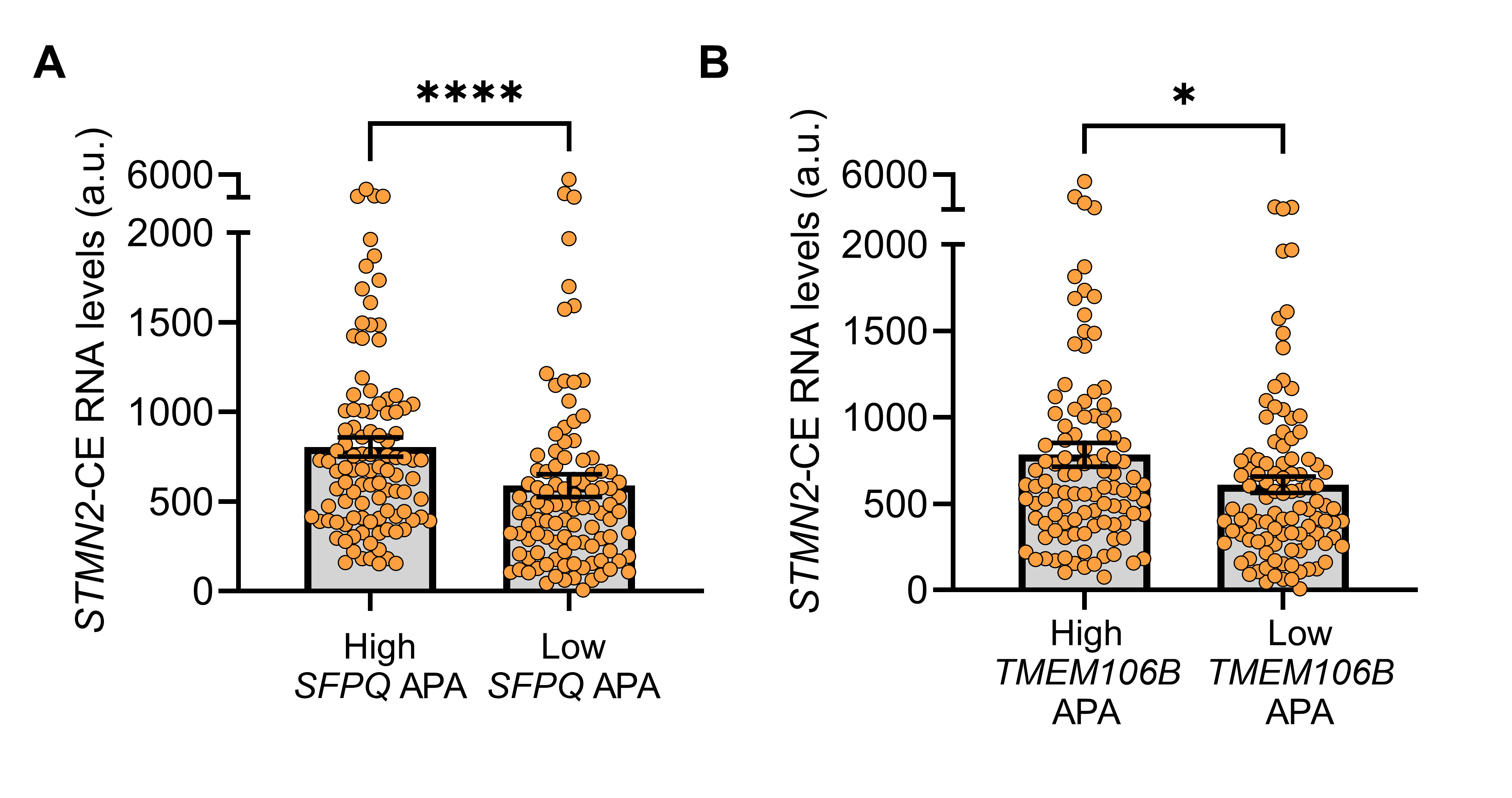

Supplement: S1 Fig — High SFPQ APA (A) and TMEM106B APA (B) have significantly higher STMN2-CE RNA when compared to their respective Low APA group (Low APA as the bottom 50%, and High APA as the top 50%, N = 104 each). Data are presented as mean ± SEM. Statistical analyses were performed by Mann–Whitney test: *P < 0.05. Data used to generate graphs can be found in S1 Data. (TIF) [file pbio.3003573.s001.tif]

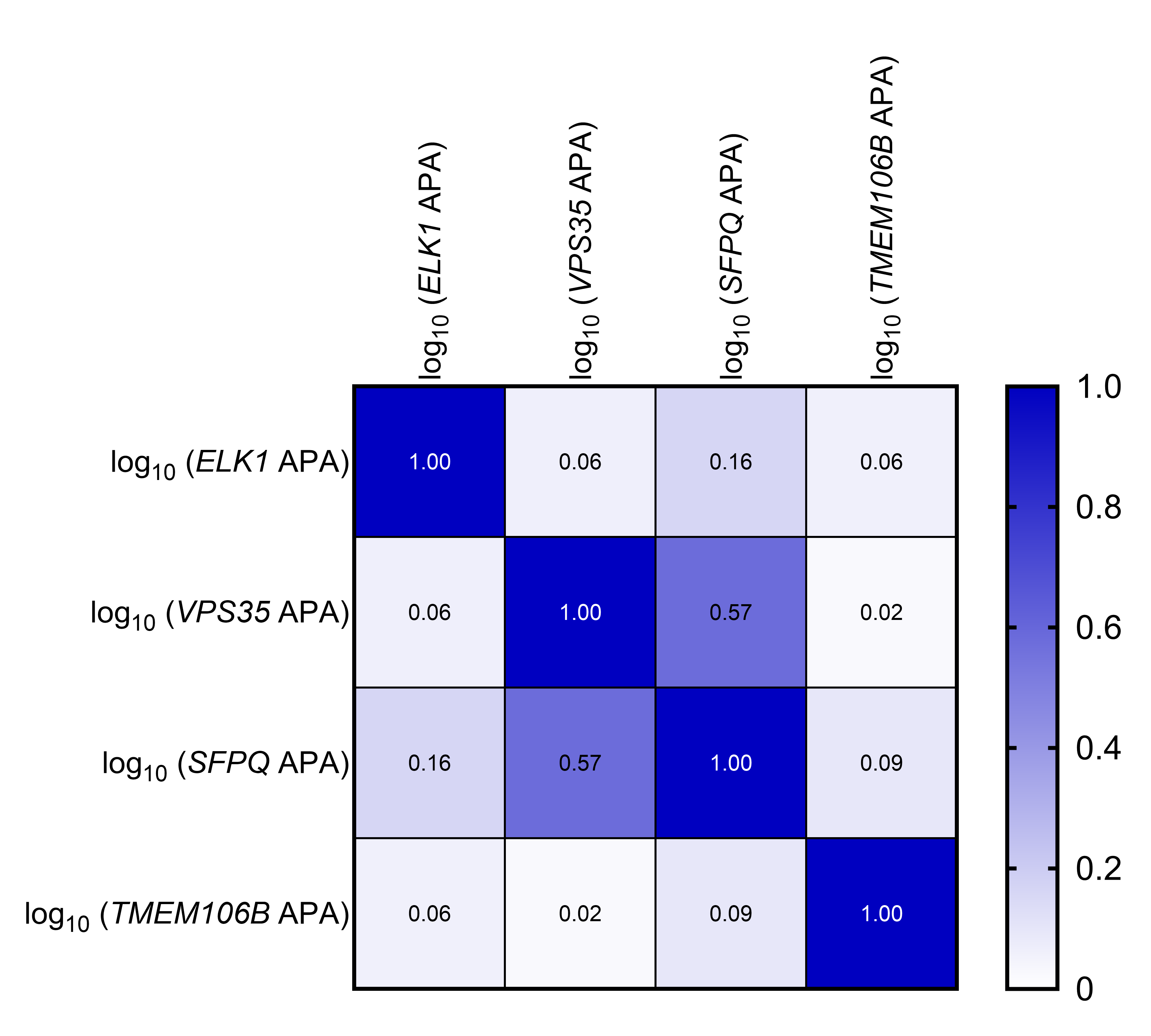

Supplement: S2 Fig — Heatmap demonstrating the pairwise APA concordance among the four targets: ELK1, VPS35, SFPQ, and TMEM106B APA using Spearman Rank correlation. A statistically significant positive association between the APA of VPS35 and SFPQ (Spearman R = 0.57, P < 0.0001), and a weak, but statistically significant positive association between the APA of ELK1 and SFPQ (Spearman R = 0.16, P = 0.017) were observed. Data used to generate graphs can be found in S1 Data. (TIF) [file pbio.3003573.s002.tif]

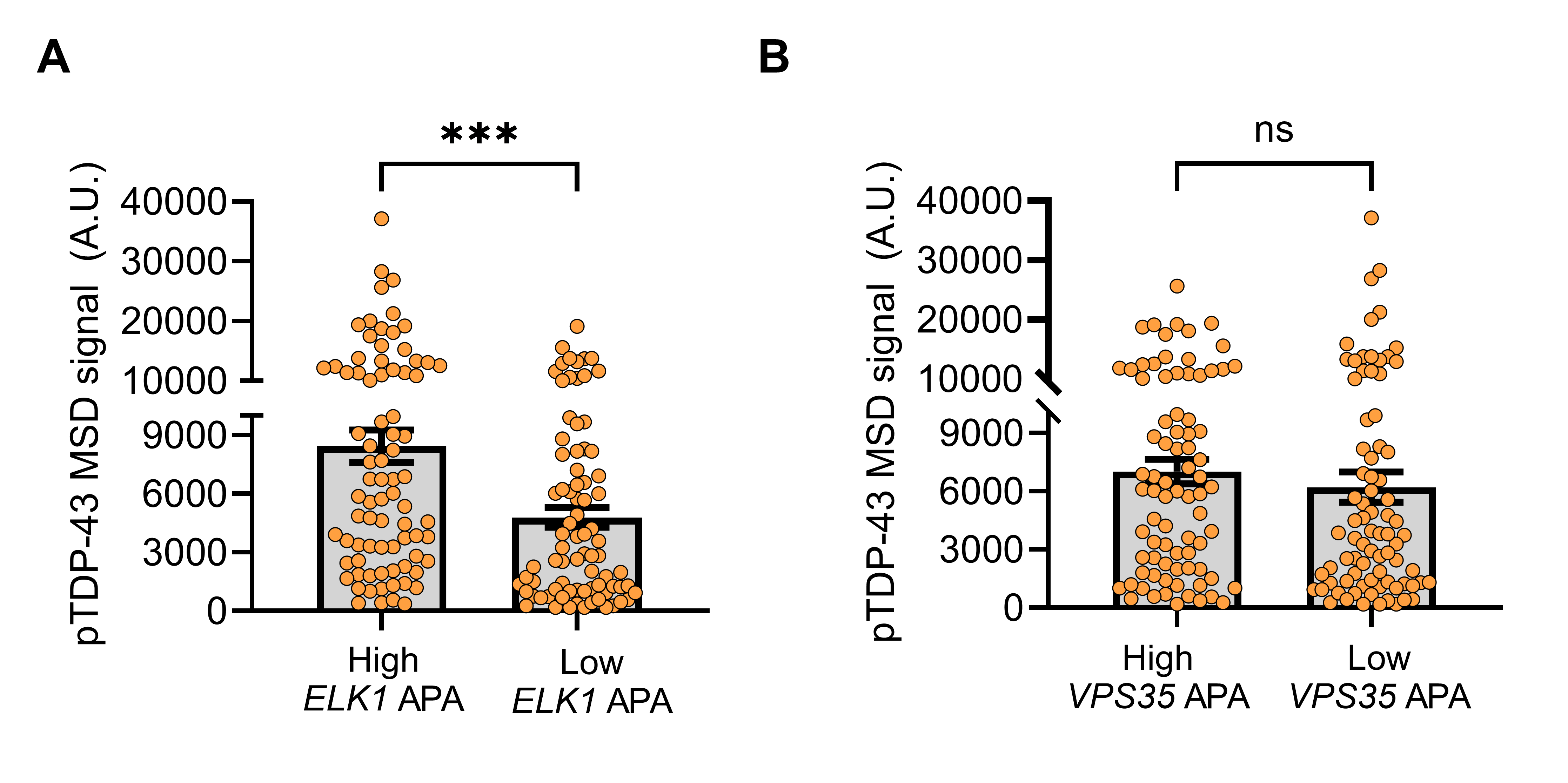

Supplement: S3 Fig — (A) When stratifying ELK1 APA by high or low APA status (Low APA as the bottom 50%, and High APA as the top 50%, N = 80 each), we observed that pTDP-43 was elevated in the high APA group compared to the low ELK1 APA. (B) No difference in pTDP-43 levels was observed between the high and low VPS35 APA groups. Data are presented as mean ± SEM. Statistical analyses were performed by Mann–Whitney test: ***P < 0.001, ns, not significant. Data used to generate graphs can be found in S1 Data. (TIF) [file pbio.3003573.s003.tif]

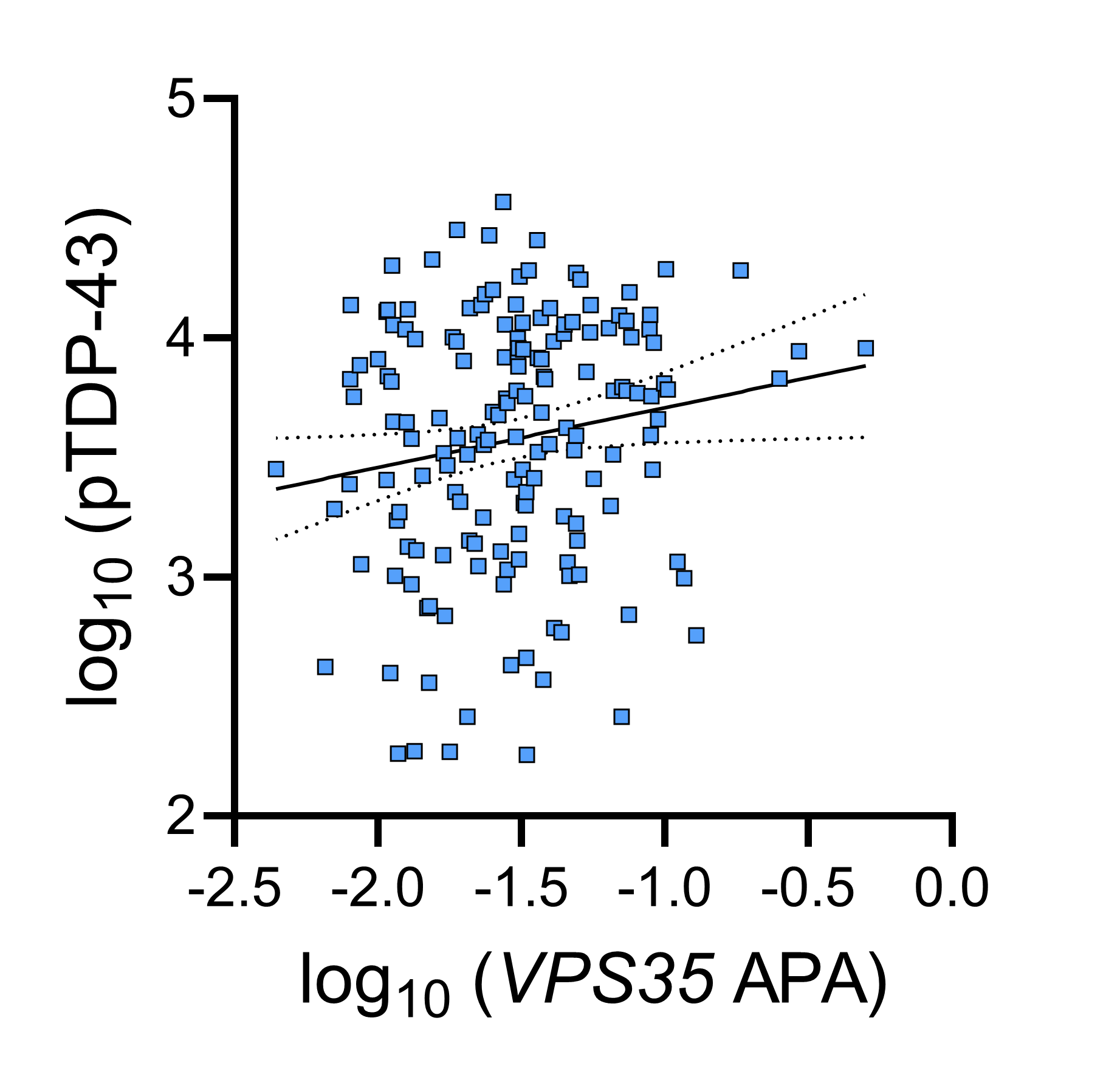

Supplement: S4 Fig — We observed a nominally significant, positive association of VPS35 APA with pTDP-43 levels (β: 0.1084, 95% CI: 0.0064 to 0.2103, P = 0.0373, S5 Table). No significant association was observed in analysis adjusting for age at death, sex, and RIN (β:0.1044, 95% CI:0.0014 to 0.2103, P = 0.0533, S5 Table). The simple linear regression fit is indicated by a solid black line, and the 95% confidence intervals (CI) are indicated by the dotted lines. Data used to generate graphs can be found in S1 Data. (TIF) [file pbio.3003573.s004.tif]

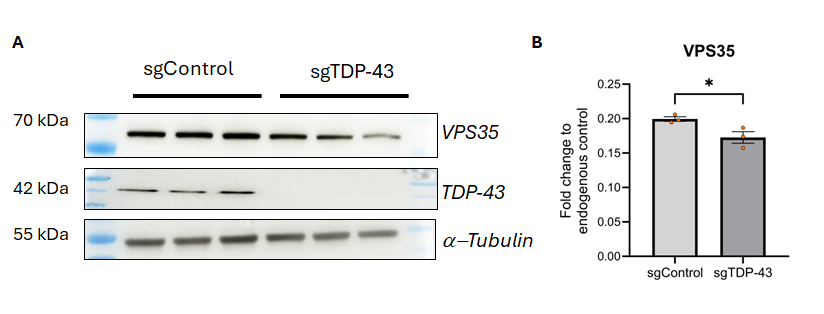

Supplement: S5 Fig — (A) Western blot image of VPS35 protein levels in iNeurons with TDP-43 knockdown (Loading control: α-tubulin). (B) Quantitation of the VPS35 protein levels. (TIF) [file pbio.3003573.s005.tif]

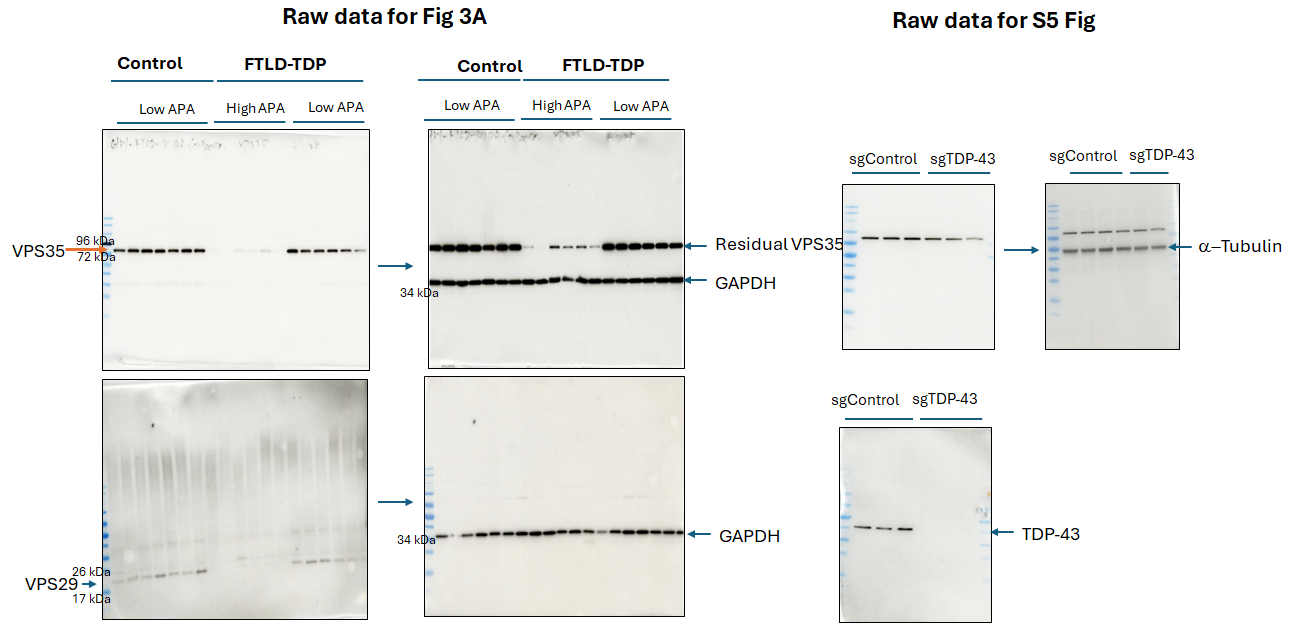

Supplement: S1 Raw Images — Raw data for Fig 3A. VPS35 and VPS29 protein levels were significantly decreased in FTLD-TDP patients with High VPS35 APA compared to FTLD-TDP patients with Low VPS35 APA and to healthy controls. RIPA soluble protein fractions were extracted from the frontal cortex of the healthy controls and FTLD-TDP patients. GAPDH: Loading control. Raw data for S5 Fig. VPS35 protein levels in iNeurons with TDP-43 knockdown. (TIF) [file pbio.3003573.s006.tif]
