# Supplementary material for: TDP-43-mediated alternative polyadenylation is associated with a reduction in VPS35 and VPS29 expression in frontotemporal dementia
Source: PLoS Biol. 2026 Jan 5;24(1):e3003573. doi: 10.1371/journal.pbio.3003573 (PMC12768243; doi:10.1371/journal.pbio.3003573)
Supplement: S1 Table — The delta_distal_PAS_usage and padj from 3′ end sequencing in TDP-43 knockdown iNeurons were described previously in Zeng and colleagues [12]. APA changes measured using 3′ end sequencing were considered significant if the adjusted P-value was <0.05. (DOCX) [file pbio.3003573.s007.docx]

S1 Table

| **Validation of targets identified by 3’ end seq in TDP-43 knockdown iNeurons in the frontal cortex of FTLD-TDP cases using qRT-PCR** | | | | | |
| --- | --- | --- | --- | --- | --- |
| **Targets identified by 3’ end seq in TDP-43 knockdown iNeurons** | | | | **qRT-PCR validation in**  **FTLD-TDP cohort** | |
| **GENE** | **APA direction** | **Delta_distal_PAS_usage** | **padj** | **Data** |  |
| *VPS35* | longer | 0.407833929 | 2.67E-89 | Current study |  |
| *VPS26B* | longer | 0.086052235 | 1.11E-38 | Current study |  |
| *ELK1* | longer | 0.643517975 | 2.63E-136 | Current study |  |
| *SFPQ* | longer | 0.191455404 | 1.44E-128 | *Zeng et al.,* Nat neurosci (2025) |  |
| *TMEM106B* | longer | 0.059235901 | 1.94E-04 | *Zeng et al.,* Nat neurosci (2025) |  |
