# Supplementary material for: TDP-43-mediated alternative polyadenylation is associated with a reduction in VPS35 and VPS29 expression in frontotemporal dementia
Source: PLoS Biol. 2026 Jan 5;24(1):e3003573. doi: 10.1371/journal.pbio.3003573 (PMC12768243; doi:10.1371/journal.pbio.3003573)
Supplement: S2 Table — The sample mean (minimum, maximum) is given for continuous variables, and number (%) of cases is provided for categorical variables. Information was unavailable for a subset of FTLD-TDP patients regarding age at onset (N = 20), disease duration (N = 19), and age at death (N = 2). (DOCX) [file pbio.3003573.s008.docx]

S2 Table

| **Patient characteristics in a postmortem cohortsof healthy controls and FTLD-TDP cases** | | |
| --- | --- | --- |
| **Variable** | **Controls (N=51)** | **FTLD-TDP (N=220)** |
| **Age at death (yrs)** | 80.01 (54.37,98.95) | 72.64 (44.47, 99.56) |
| **Sex (female)** | 20 (39.2%) | 104 (47.3%) |
| **Presence of motor neuron disease** | NA | 57 (25.9%) |
| **Disease duration (yrs)** | NA | 7.61 (1.0,25.0) |
| **Age at onset (yrs)** | NA | 65.05 (39.98, 89.62) |
| The sample mean (minimum, maximum) is given for continuous variables, and number of cases (%) is provided for categorical variables. Information was unavailable for a subset of FTLD-TDP patients regarding age at onset (N=20), disease duration (i.e., time from symptom onset to death) (N=19), and age at death (N=2). | | |
