# Supplementary material for: TDP-43-mediated alternative polyadenylation is associated with a reduction in VPS35 and VPS29 expression in frontotemporal dementia
Source: PLoS Biol. 2026 Jan 5;24(1):e3003573. doi: 10.1371/journal.pbio.3003573 (PMC12768243; doi:10.1371/journal.pbio.3003573)
Supplement: S3 Table — CI, confidence interval; Regression coefficients, 95% CIs, and P-values result from unadjusted linear regression models or linear regression models adjusted for sex, RIN, and age at death where APA and STMN2-CE RNA levels were considered on the base 10 logarithmic scale. STMN2-CE RNA levels were measured on a subset of FTLD-TDP cases, N = 206. P-values <0.0125 are considered statistically significant after correcting for multiple testing. Significance is denoted by bolded text. (DOCX) [file pbio.3003573.s009.docx]

S3 Table

| **TDP-43-mediated 3’UTR lengthening associates with *STMN2-CE* RNA in the frontal cortex of FTLD-TDP cases** | | | | |
| --- | --- | --- | --- | --- |
|  | **Unadjusted analyses** | | **Multivariable analyses**  **(adjusted for age at death, sex and RIN)** | |
| **Gene with TDP-43-mediated APA** | **Regression coefficient**  **(95% CI)** | **P-value** | **Regression coefficient**  **(95% CI)** | **P-value** |
| ***ELK1*** | 0.2077 (0.1065 to 0.3090) | **<0.0001** | 0.1827 (0.0764 to 0.2888) | **0.0008** |
| ***VPS35*** | 0.2263 (0.1041 to 0.3484) | **0.0003** | 0.1919 (0.0672 to 0.3165) | **0.0027** |
| ***SFPQ*** | 0.3526 (0.2120 to 0.4932) | **<0.0001** | 0.3444 (0.1985 to 0.4904) | **<0.0001** |
| ***TMEM106B*** | 0.0891 (0.0184 to 0.1598) | 0.0137 | 0.0958 (0.0204 to 0.1713) | 0.0130 |
| CI: confidence interval. | | | | |
