# Supplementary material for: TDP-43-mediated alternative polyadenylation is associated with a reduction in VPS35 and VPS29 expression in frontotemporal dementia
Source: PLoS Biol. 2026 Jan 5;24(1):e3003573. doi: 10.1371/journal.pbio.3003573 (PMC12768243; doi:10.1371/journal.pbio.3003573)
Supplement: S4 Table — CI, confidence interval; Regression coefficients, 95% CIs, and P-values result from unadjusted linear regression models or linear regression models adjusted for sex, RIN, and age at death where APA and HDGFL2-CE protein levels were considered on the base 10 logarithmic scale. P-values <0.0125 are considered statistically significant after correcting for multiple testing. Significance is denoted by bolded text. (DOCX) [file pbio.3003573.s010.docx]

S4 Table

| **TDP-43-mediated 3’UTR lengthening of ELK1 is associated with HDGFL2-CE protein in the frontal cortex of FTLD-TDP cases** | | | | |
| --- | --- | --- | --- | --- |
|  | **Unadjusted analyses** | | **Multivariable analyses**  **(adjusted for age at death, sex and RIN)** | |
| **Gene with TDP-43-mediated APA** | **Regression coefficient (95% CI)** | **P-value** | **Regression coefficient (95% CI)** | **P-value** |
| ***ELK1*** | 0.2300 (0.1574 to 0.3026) | **<0.0001** | 0.2035 (0.1266 to 0.2803) | **<0.0001** |
| ***VPS35*** | 0.0554 (-0.0432 to 0.1541) | 0.2693 | 0.1117 (0.0127 to 0.2107) | 0.0271 |
| ***SFPQ*** | -0.0371 (-0.1488 to 0.0745) | 0.5131 | 0.0052 (-0.1093 to 0.1197) | 0.9283 |
| ***TMEM106B*** | -0.0346 (-0.0885 to 0.0193) | 0.2072 | -0.0432 (-0.1005 to 0.0142) | 0.1394 |
| CI: confidence interval. | | | | |
