# Supplementary material for: TDP-43-mediated alternative polyadenylation is associated with a reduction in VPS35 and VPS29 expression in frontotemporal dementia
Source: PLoS Biol. 2026 Jan 5;24(1):e3003573. doi: 10.1371/journal.pbio.3003573 (PMC12768243; doi:10.1371/journal.pbio.3003573)
Supplement: S5 Table — CI, confidence interval; Regression coefficients, 95% CIs, and P-values result from unadjusted linear regression models or linear regression models adjusted for sex, RIN, and age at death where APA levels were considered on the base 10 logarithmic scale. pTDP-43 levels were measured on a subset of FTLD-TDP cases, N = 160. P-values <0.0125 are considered statistically significant after correcting for multiple testing. Significance is denoted by bolded text. (DOCX) [file pbio.3003573.s011.docx]

S5 Table

| **TDP-43-mediated 3’UTR lengthening of *ELK1* is associated with pTDP-43 burden in the frontal cortex of FTLD-TDP cases** | | | | |
| --- | --- | --- | --- | --- |
|  | **Unadjusted analyses** | | **Multivariable analyses**  **(adjusted for age at death, sex and RIN)** | |
|  | Regression coefficient (95% CI) | P-value | Regression coefficient  (95% CI) | P-value |
| ***ELK1*** | 0.1246 (0.0491 to 0.2001) | **0.0014** | 0.0927 (0.0127 to 0.1728) | 0.0234 |
| ***VPS35*** | 0.1084 (0.0064 to 0.2103) | 0.0373 | 0.1044 (-0.0014 to 0.2103) | 0.0533 |
| ***SFPQ*** | -0.0133 (-0.1378 to 0.1111) | 0.8326 | -0.0102 (-0.1402 to 0.1199) | 0.8775 |
| ***TMEM106B*** | -0.0290 (-0.0842 to 0.0262) | 0.3008 | -0.0186 (-0.0777 to 0.0403) | 0.5331 |
| CI: confidence interval. | | | | |
