# Supplementary material for: TDP-43-mediated alternative polyadenylation is associated with a reduction in VPS35 and VPS29 expression in frontotemporal dementia
Source: PLoS Biol. 2026 Jan 5;24(1):e3003573. doi: 10.1371/journal.pbio.3003573 (PMC12768243; doi:10.1371/journal.pbio.3003573)
Supplement: S6 Table — CI, confidence interval; Regression coefficients, 95% CIs, and P-values result from unadjusted and adjusted linear regression models where APA levels were considered on the base 10 logarithmic scale. P-values <0.0125 are considered statistically significant after correcting for multiple testing. Multivariable analysis associations of APA with age at onset was corrected for sex, RIN, and presence of MND. Multivariable analysis associations of APA with disease duration (time between age of onset and time) were corrected for sex, RIN, age at onset, and presence of MND. Significance is denoted by bolded text. (DOCX) [file pbio.3003573.s012.docx]

S6 Table

| **TDP-43-mediated 3’UTR lengthening of *ELK1* is associated with clinical characteristics in the frontal cortex of FTLD-TDP cases** | | | | |
| --- | --- | --- | --- | --- |
|  | **Unadjusted analysis** | | **Multivariable analysis** | |
|  | Regression coefficient  (95% CI) | P-value | Regression coefficient  (95% CI) | P-value |
| **Association of APA with age at onset**  **(adjusted for sex, RIN and presence of MND)** | | | | |
| *ELK1* | -0.0074 (-0.0117 to -0.0031) | **0.0008** | -0.0077 (-0.0121 to -0.0033) | **0.0006** |
| *VPS35* | 0.0052 (0.0002 to 0.0102) | 0.0405 | 0.0033 (-0.0014 to 0.0081) | 0.1649 |
| *SFPQ* | 0.0054 (-0.0002 to 0.0112) | 0.0619 | 0.0038 (-0.0017 to 0.0094) | 0.1734 |
| *TMEM106B* | -0.0006 (-0.0034 to 0.0021) | 0.6587 | -0.0002 (-0.0031 to 0.0026) | 0.8624 |
| **Association of APA with disease duration**  **(adjusted for sex, RIN, age at onset and presence of MND)** | | | | |
| *ELK1* | -0.0085 (-0.0175 to 0.0005) | 0.0635 | -0.0132 (-0.0230 to -0.0034) | **0.0082** |
| *VPS35* | 0.0032 (-0.0072 to 0.0136) | 0.5452 | -0.0025 (-0.0134 to 0.0082) | 0.6380 |
| *SFPQ* | -0.0003 (-0.0122 to 0.0116) | 0.9596 | -0.0052 (-0.0178 to 0.0072) | 0.4072 |
| *TMEM106B* | -0.0022 (-0.0080 to 0.0036) | 0.4573 | -0.0086 (-0.0073 to 0.0056) | 0.7942 |
| CI: confidence interval. | | | | |
