# Supplementary material for: TDP-43-mediated alternative polyadenylation is associated with a reduction in VPS35 and VPS29 expression in frontotemporal dementia
Source: PLoS Biol. 2026 Jan 5;24(1):e3003573. doi: 10.1371/journal.pbio.3003573 (PMC12768243; doi:10.1371/journal.pbio.3003573)
Supplement: S7 Table — CI, confidence interval; Regression coefficients, 95% CIs, and P-values result from unadjusted linear regression models or linear regression models adjusted for sex, RIN, and age at death where VPS35 RNA and protein levels were considered on the base 10 logarithmic scale. Sample size of N = 192 for VPS35 protein levels and N = 153 for VPS29 protein levels were used depending on sample availability. P-values <0.025 are considered statistically significant after correcting for multiple testing. Significance is denoted by bolded text. (DOCX) [file pbio.3003573.s013.docx]

S7 Table

| ***VPS35* 3’UTR lengthening correlates with VPS35 and VPS29 protein levels in the frontal cortex of FTLD-TDP cases** | | | | |
| --- | --- | --- | --- | --- |
|  | **Unadjusted analysis** | | **Multivariable analysis**  **(adjusted for age at death, sex and RIN)** | |
| Associations with *VPS35* APA | Regression coefficient (95% CI) | P-value | Regression coefficient (95% CI) | P-value |
| VPS35 protein | -0.2307 (-0.3158 to -0.1455) | **<0.0001** | -0.2109 (-0.3092 to -0.1127) | **<0.0001** |
| VPS29 protein | -0.4257 (-0.5738 to -0.2777) | **<0.0001** | -0.3928 (-0.5639 to -0.2217) | **<0.0001** |
| CI: confidence interval. | | | | |
