# Supplementary material for: TDP-43-mediated alternative polyadenylation is associated with a reduction in VPS35 and VPS29 expression in frontotemporal dementia
Source: PLoS Biol. 2026 Jan 5;24(1):e3003573. doi: 10.1371/journal.pbio.3003573 (PMC12768243; doi:10.1371/journal.pbio.3003573)
Supplement: S8 Table — β, regression coefficient; CI, confidence interval; AIC, Akaike Information Criterion. β, 95% CIs, AICs, and P-values result from unadjusted linear regression models or linear regression models adjusted for either age at death and sex or age at death, sex, and presence of MND where pTDP-43 levels were considered on the base 10 logarithmic scale. Beta coefficients can be interpreted as the change in log10(pTDP-43) levels given a one-unit increase in VPS35 protein levels in the insoluble fraction. Lower AIC scores indicate that a model produces a better fit to the data. After applying Bonferroni correction, adjusted p-values <0.0167 were determined to be significant. Significance is denoted by bolded text. (DOCX) [file pbio.3003573.s014.docx]

S8 Table

| **VPS35 protein is associated with pTDP-43 burden in the frontal cortex of FTLD-TDP cases** | | | | | |
| --- | --- | --- | --- | --- | --- |
| **Associations of VPS35 protein with pTDP-43** | β **coefficient** | **95 % CI Lower** | **95 % CI Upper** | **P-value** | **AIC** |
| Unadjusted | -0.0026 | -0.0048 | -0.0004 | 0.0207 | 152.2774 |
| Adjusted by age at death and sex | -0.0029 | -0.0050 | -0.0009 | **0.0061** | 138.3665 |
| Adjusted by age at death, sex and  presence of MND | -0.0026 | -0.0047 | -0.0005 | **0.0147** | 138.3281 |
| CI: confidence interval; AIC = Akaike Information Criterion. | | | | | |
