# Supplementary material for: TDP-43-mediated alternative polyadenylation is associated with a reduction in VPS35 and VPS29 expression in frontotemporal dementia
Source: PLoS Biol. 2026 Jan 5;24(1):e3003573. doi: 10.1371/journal.pbio.3003573 (PMC12768243; doi:10.1371/journal.pbio.3003573)
Supplement: S9 Table — β, regression coefficient; CI, confidence interval; AIC, Akaike Information Criterion. β, 95% CIs, AICs, and P-values result from unadjusted linear regression models or linear regression models adjusted for either age at death and sex or age at death, sex, and presence of MND. VPS35 protein shows a significant inverse association with STMN2-CE RNA levels in the FTLD-TDP postmortem brain. Significance is denoted by bolded text. (DOCX) [file pbio.3003573.s015.docx]

S9 Table

| **VPS35 protein is associated with *STMN2-CE* RNA in the frontal cortex of FTLD-TDP cases** | | | | | |
| --- | --- | --- | --- | --- | --- |
| **Associations with VPS35 protein with *STMN2-CE* RNA** | β **coefficient** | **95 % CI Lower** | **95 % CI Upper** | **P-value** | **AIC** |
| Unadjusted | -7.9420 | -10.8033 | -5.0806 | **1.39e-07** | 1378.876 |
| Adjusted by age at death and sex | -5.5846 | -8.7868 | -2.3825 | **0.0007** | 1355.825 |
| Adjusted by age at death, sex and  presence of MND | -5.3714 | -8.6963 | -2.0465 | **0.0017** | 1357.584 |
| b:CI: confidence interval; AIC = Akaike Information Criterion. | | | | | |
